# Deep Learning–based Automatic Lung Segmentation on Multiresolution CT Scans from Healthy and Fibrotic Lungs in Mice

**DOI:** 10.1148/ryai.210095

**Published:** 2022-01-12

**Authors:** Francesco Sforazzini, Patrick Salome, Mahmoud Moustafa, Cheng Zhou, Christian Schwager, Katrin Rein, Nina Bougatf, Andreas Kudak, Henry Woodruff, Ludwig Dubois, Philippe Lambin, Jürgen Debus, Amir Abdollahi, Maximilian Knoll

**Affiliations:** From the Clinical Cooperation Unit Radiation Oncology, German Cancer Research Center (DKFZ), Im Neuenheimer Feld 280, 69120 Heidelberg, Germany (F.S., P.S., M.M., C.Z., C.S., K.R., N.B., A.K., J.D., A.A., M.K.); Department of Radiation Oncology (F.S., P.S., M.M., C.Z., C.S., K.R., N.B., A.K., J.D., A.A., M.K.) and National Center for Tumor Diseases (NCT) (F.S., P.S., M.M., C.Z., C.S., K.R., N.B., J.D., A.A., M.K.), Heidelberg University Hospital (UKHD), Heidelberg, Germany; German Cancer Consortium (DKTK) Core Center Heidelberg, Heidelberg, Germany (F.S., P.S., M.M., C.Z., C.S., K.R., J.D., A.A., M.K.); National Center for Radiation Oncology (NCRO), Heidelberg Institute for Radiation Oncology (HIRO), Heidelberg, Germany (F.S., P.S., M.M., C.Z., C.S., K.R., N.B., A.K., J.D., A.A., M.K.); Heidelberg Ion-Beam Therapy Center (HIT), Heidelberg, Germany (F.S., P.S., M.M., C.Z., C.S., K.R., N.B., J.D., A.A., M.K.); Department of Clinical Pathology, Suez Canal University, Ismailia, Egypt (M.M.); Department of Radiation Oncology, Nanfang Hospital, Southern Medical University, Guangzhou, China (C.Z.); and The D-Laboratory and the M-Laboratory, Department of Precision Medicine, GROW-School for Oncology, Maastricht University, Maastricht, the Netherlands (H.W., L.D., P.L.).

**Keywords:** Deep Learning, Lung Fibrosis, Radiation Therapy, Segmentation, Animal Studies, CT, Thorax, Lung

## Abstract

**Purpose:**

To develop a model to accurately segment mouse lungs with varying levels of fibrosis and investigate its applicability to mouse images with different resolutions.

**Materials and Methods:**

In this experimental retrospective study, a U-Net was trained to automatically segment lungs on mouse CT images. The model was trained (*n* = 1200), validated (*n* = 300), and tested (*n* = 154) on longitudinally acquired and semiautomatically segmented CT images, which included both healthy and irradiated mice (group A). A second independent group of 237 mice (group B) was used for external testing. The Dice score coefficient (DSC) and Hausdorff distance (HD) were used as metrics to quantify segmentation accuracy. Transfer learning was applied to adapt the model to high-spatial-resolution mouse micro-CT segmentation (*n* = 20; group C [*n* = 16 for training and *n* = 4 for testing]).

**Results:**

The trained model yielded a high median DSC in both test datasets: 0.984 (interquartile range [IQR], 0.977–0.988) in group A and 0.966 (IQR, 0.955–0.972) in group B. The median HD in both test datasets was 0.47 mm (IQR, 0–0.51 mm [group A]) and 0.31 mm (IQR, 0.30–0.32 mm [group B]). Spatially resolved quantification of differences toward reference masks revealed two hot spots close to the air-tissue interfaces, which are particularly prone to deviation. Finally, for the higher-resolution mouse CT images, the median DSC was 0.905 (IQR, 0.902–0.929) and the median 95th percentile of the HD was 0.33 mm (IQR, 2.61–2.78 mm).

**Conclusion:**

The developed deep learning–based method for mouse lung segmentation performed well independently of disease state (healthy, fibrotic, emphysematous lungs) and CT resolution.

**Keywords:** Deep Learning, Lung Fibrosis, Radiation Therapy, Segmentation, Animal Studies, CT, Thorax, Lung

*Supplemental material is available for this article*.

Published under a CC BY 4.0 license.

## Graphical abstract

**Figure:**
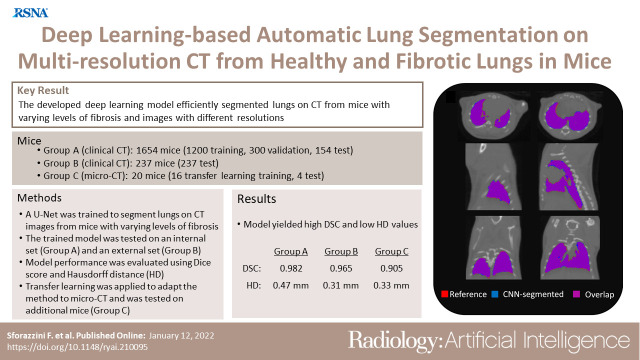

**Summary**

In this experimental murine study, the developed deep learning model efficiently segmented lungs on CT images in mice with varying levels of fibrosis and on images with different resolutions.

**Key Points**

■ The developed deep learning–based segmentation model was trained and validated on CT images from 1500 mice and then tested on an internal (*n* = 154) and external (*n* = 237) dataset.
■ On the internal test set, the model yielded a median Dice score coefficient (DSC) and Hausdorff distance (HD) of 0.984 (interquartile range [IQR], 0.977–0.988) and 0.47 mm (IQR, 0–0.51 mm), respectively.
■ On the external test dataset, the median DSC was 0.966 (IQR, 0.955–0.972), and the median HD was 0.31 mm (IQR, 0.30–0.32 mm).
■ Finally, the applicability of the model to segment lungs from high-resolution mouse micro-CT was investigated after transfer learning, with a median DSC of 0.905 and a median 95th percentile of HD of 0.33 mm.

## Introduction

Radiation therapy is an integral part of cancer treatment and aims for precise, high-dose irradiation of tumors to achieve a high probability of tumor control. Normal tissue must be spared to avoid tissue toxicity and to lower the rate of potential complications. For treatments within the thoracic region, it is important to account for the fact that the lung, together with the heart and spinal cord, is a dose-limiting organ. The main relevant adverse effects of irradiation are pneumonitis (short-term) and lung fibrosis (long-term).

Rodent animal models are established for the preclinical study of pathophysiologic processes involved in the development of lung fibrosis, which can be monitored with clinical CT or micro-CT. Lung volume or specific metrics, such as the fibrosis index, can be used as a surrogate for lung fibrosis (1). However, such analyses rely on accurate lung segmentation, irrespective of the degree of fibrosis or otherwise altered lung parenchyma (eg, emphysema).

Segmentation can be performed by different methods, with manual segmentation having the disadvantages of being time-consuming and lacking consensus guidelines for segmentation (eg, inclusion and exclusion of the bronchial system); such variability may impair interpretability and reproducibility. Semiautomatic approaches use Hounsfield unit thresholds to automatically identify low-attenuation lung parenchyma followed by manual curation (1,2). Finally, atlas-based registration (3,4) and multiorgan segmentation following injection of contrast agent (5) have been described for micro-CT data. Their dependency on specific CT scanner and/or acquisition setups impair generalizability, however. Furthermore, methods are usually only tested on healthy mice; thus, performance in fibrotic and emphysematous lungs is uncertain.

Artificial intelligence, in particular deep learning (6,7), has been successfully applied to several medical imaging tasks (7). For medical image segmentation, the development of the U-Net helped to advance these tasks (8). This network and its variations have been applied to multiple medical imaging segmentation tasks (8–10).

In this study, we aimed to develop and test a deep learning–based method to automatically segment mouse lungs with varying levels of fibrosis from clinical CT images. We also explored the applicability of a transfer learning approach to obtain lung segmentation using the proposed network from high-spatial-resolution mouse micro-CT images.

## Materials and Methods

### Study Design

**Figure 1:**
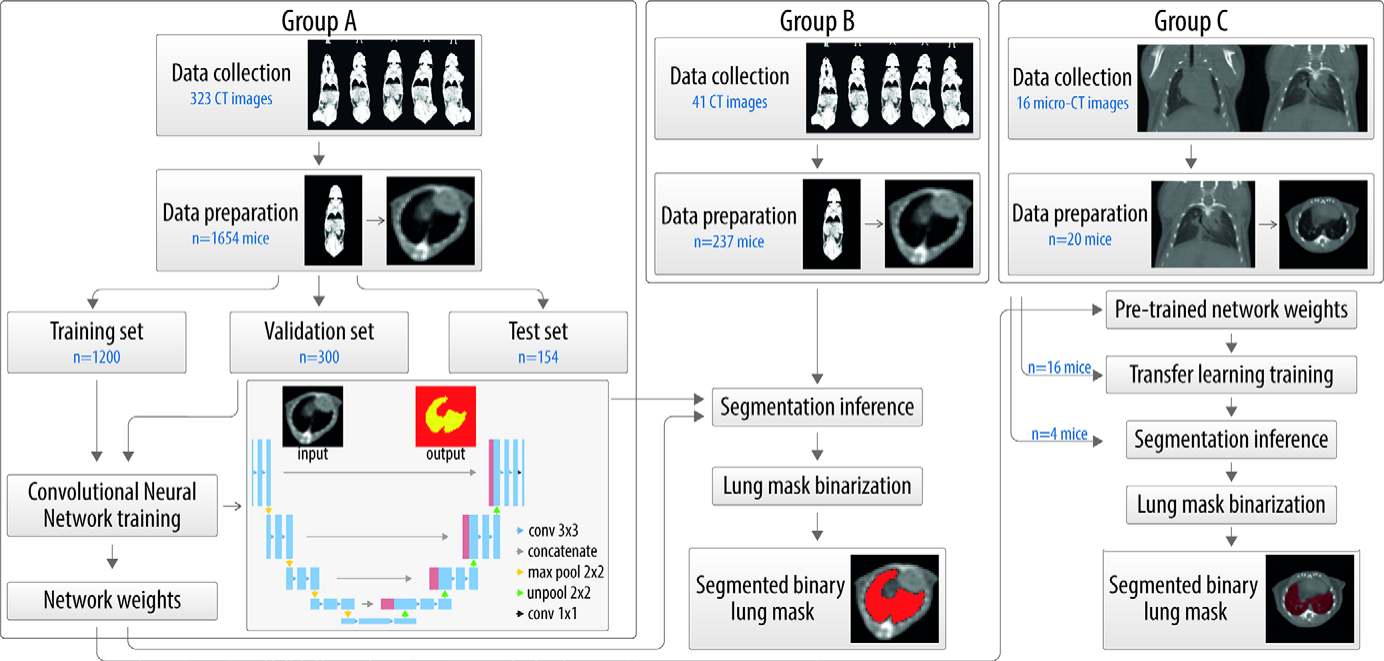
Overview of the workflow used for training, validation, and testing a U-Net convolutional neural network for lung segmentation. The main mouse CT group (group A [left]), an independent group of mice (group B [center]), and a separate group of healthy mice (group C [right]) are shown. conv = convolution, max pool = max pooling, unpool = unpooling.

A total of 323 CT images (group A; acquired at our institution between 2016 and 2017, with images used for training, validation, and testing) and 41 CT images (group B; acquired in 2014 with different resolution with regard to group A, with images used only for testing) were retrospectively collected from multiple mouse trials focused only on studying fibrosis and not on the development of a segmentation model (1,2). The number of irradiated and healthy mice and the age ranges for the two groups are reported in Tables E1 and E2 (supplement). No further selection criteria were applied. These trials contained both healthy and irradiated (modalities: photon or particles) mice with different levels of fibrosis. Additionally, 16 CT images (group C) from healthy mice (age, 12 weeks) were retrospectively collected from an existing project from another institution (11). The CT images contained multiple mice per image, as explained later in this section. Information about the three groups is summarized in the Table. Animal care ethical approvals are reported in the respective original studies. An overview of the groups and methods is shown in Figure 1.

**Table:**
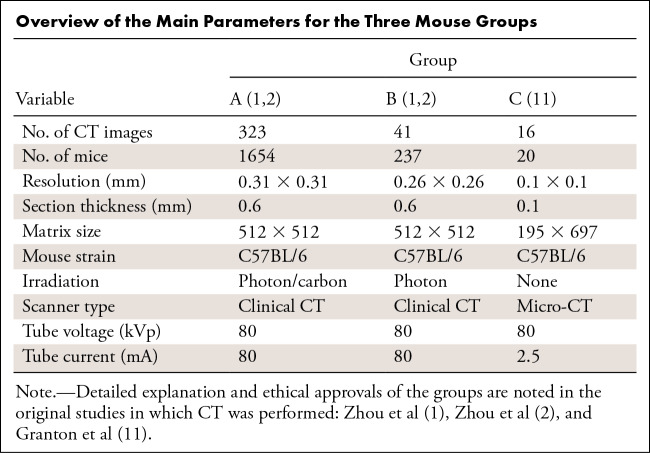
Overview of the Main Parameters for the Three Mouse Groups

### Image Acquisition

For groups A and B, CT images were acquired using a clinical PET/CT scanner (Biograph mCT, Siemens). The standard protocol used for the CT portion of PET/CT was as follows: 80 kV with 80 mAs, a pitch of 0.6 mm, section thickness of 0.6 mm, and acquisition time of 32 second (1).

For group C, CT images were acquired using a small animal microirradiator (micro-IR, X-RAD 225Cx; Precision X-Ray) with integrated micro-CT. A high-resolution 80-kVp, 2.5-mA imaging protocol filtered with an acquisition rate of 5 frames per second was used for all imaging (11).

### Data Partition

A total of 291 CT images (90%) from group A were used for training and validation. These 291 images were split into five different folds using k-fold cross-validation (training and validation ratio of 80% and 20%, respectively). The remaining 32 CT images were used for evaluation (testing). Group B was used exclusively for testing.

A total of 80% of the CT images in group C (*n* = 13) were used to retrain the model using the transfer learning approach. The remaining three CT images were used for testing.

### Data Preparation

All the CT images were converted from Digital Imaging and Communications in Medicine format to NRRD format using the Medical Imaging Interaction Toolkit (12).

The CT images contained four to six mice each; thus, the first step was to crop each CT scan to obtain CT image data from a single mouse. Coordinates of the lung edges were identified from the reference mask and were used to find the *x, y*, and *z* coordinates to crop the corresponding CT image. This cropping resulted in a total of 1654 mice in group A (1500 mice for training and validation and 154 for testing) and 237 mice in group B. Cropped images were resized to achieve an isotropic resolution of 0.35 mm. The reference lung masks (ground truth) were segmented semiautomatically, as described by Zhou and colleagues (1,2), for both fibrotic and normal lungs. Briefly, each CT was thresholded between −900 HU and −100 HU to obtain an approximate lung segmentation. Subsequently, incorrectly assigned voxels, mostly in the trachea and primary bronchi areas, were manually removed.

The CT images from group C contained one or two mice each. Images with two mice were cropped as described previously. The total number of mice in group C was 20 (16 used to retrain the model, and four used for testing). Each cropped CT image was then resampled to an isotropic resolution of 0.2 mm. Ground truth lung masks were again segmented semiautomatically.

Finally, given the two-dimensional architecture of the proposed method, each axial section in the three-dimensional images from all groups was extracted, and the intensity was normalized between zero and 1.

### Convolutional Neural Network Architecture

The convolutional neural network (CNN) was implemented with a two-dimensional U-Net architecture consisting of one encoder, which allows capturing of the global structure of the data, and one decoder, which allows a fine-grained localization. The details of the network structure are reported in Figure E1 (supplement).

The network was trained five times, each time on a different fold, using the Adam optimization algorithm and a learning rate of 0.0001. Binary cross-entropy was used as the loss function and Dice score coefficient (DSC) as the metric. The batch size was fixed at 50 for training and validation. The number of epochs was set to 30. The total model training time was approximately 15 hours on a workstation equipped with Ubuntu (version 18.04; *https://releases.ubuntu.com/18.04/*), as well as an Intel Xeon Processor R with eight cores and 16 GB of RAM and a GeForce GTX 1060 graphics card (NVIDIA; 6 GB).

The inference was run five times, using one different set of weights each time. The final probability lung mask was calculated as the median. Both hard thresholding (0.5) and Otsu thresholding were tested to obtain the final binary lung mask. The average inference time for one mouse was 0.31 second.

To apply this network to segment lungs from mouse CT images in group C, a transfer learning approach was implemented. In particular, the encoder was frozen during the network retraining and only the decoder was further trained; this was done to enable accurate lung segmentation.

### Statistical Analysis

All the analyses described below were performed using NumPy (version 1.17.0) and MedPy (version 0.4.0), in Python (version 3.7; Python Software Foundation).

***Network performance.—***The DSC and the 95th percentile of the Hausdorff distance (HD) were used as metrics to quantify the segmentation accuracy, in terms of spatial overlap and shape mismatch (see Appendix E1 [supplement] for the definitions of DSC and HD).

***Spatial mismatch quantification.—***To find areas in the lung more sensitive to different classification, all 154 mice in the main test set were registered to a common space and then tested for deviation.

For each voxel in the difference image between the reference lung mask and the CNN segmented mask, a z-score was calculated across mice to find where the CNN segmentations more consistently deviated from the manual segmentations. See Figure E2 and Appendix E2 (supplement) for details.

***Similarity of approaches.—***To justify the use of the lung masks segmented using the proposed approach instead of those semiautomatically contoured, we compared the fibrosis indices (1,2) and the histograms of the Hounsfield units within the lungs calculated using both masks.

### Model Availability

This pipeline is available at GitHub (*https://github.com/TransRadOnc-HIT/lung_segmentation.git*).

## Results

### Network Performance

The workflow described in the present analyses, with the corresponding data, is outlined in Figure 1.

**Figure 2:**
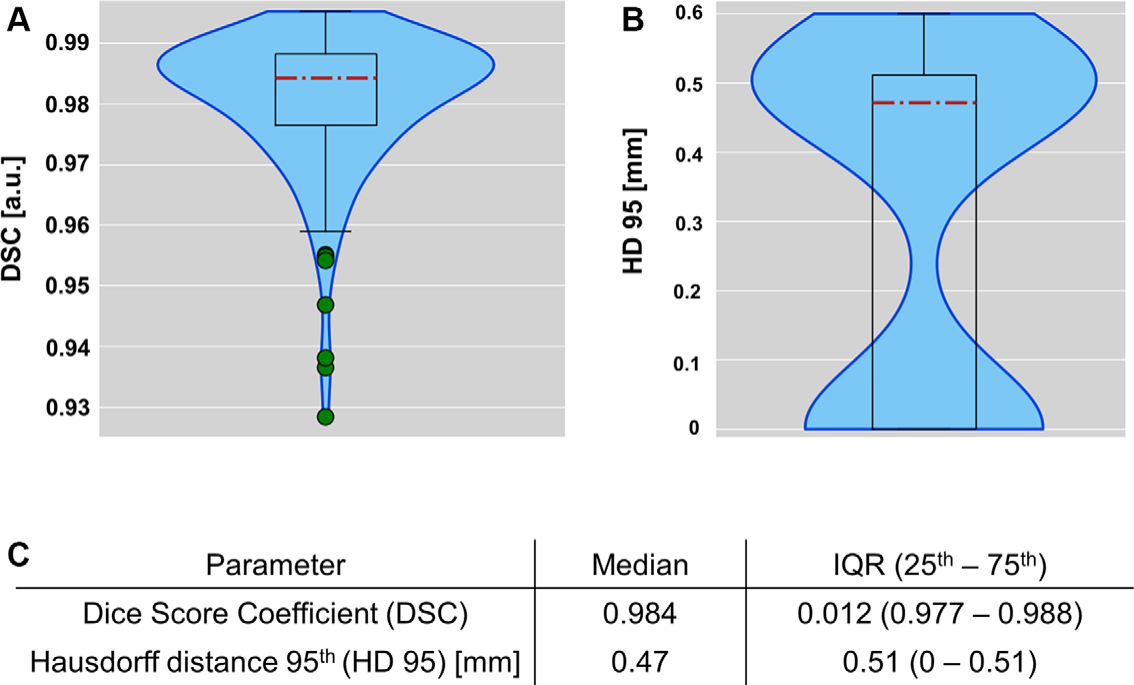
Performance of the automatic lung segmentation. Violin plots with overlaid box plots show **(A)** Dice score coefficients (DSC) and **(B)** Hausdorff distances (HD) calculated on the test set (*n* = 154 mice from group A). **(C)** Exact numeric values. a.u. = arbitrary unit, IQR = interquartile range.

A first evaluation of the proposed CNN was performed on the test dataset from group A (*n* = 154 mice). On this test set, our algorithm yielded a median DSC of 0.984, with an interquartile range (IQR) between 0.977 and 0.988 (Fig 2). The corresponding median HD (95th percentile) was 0.47 mm (IQR, 0–0.51 mm), as shown in Figure 2. The DSC and HD were also calculated using CNN lung masks binarized with a hard probability threshold of 0.5. In this case, the median DSC was 0.982 (IQR, 0.976–0.988) and the median HD was 0.47 mm (IQR, 0–0.51 mm). Given the slightly higher performance of the Otsu thresholding method, it was chosen as the binarization method.

**Figure 3:**
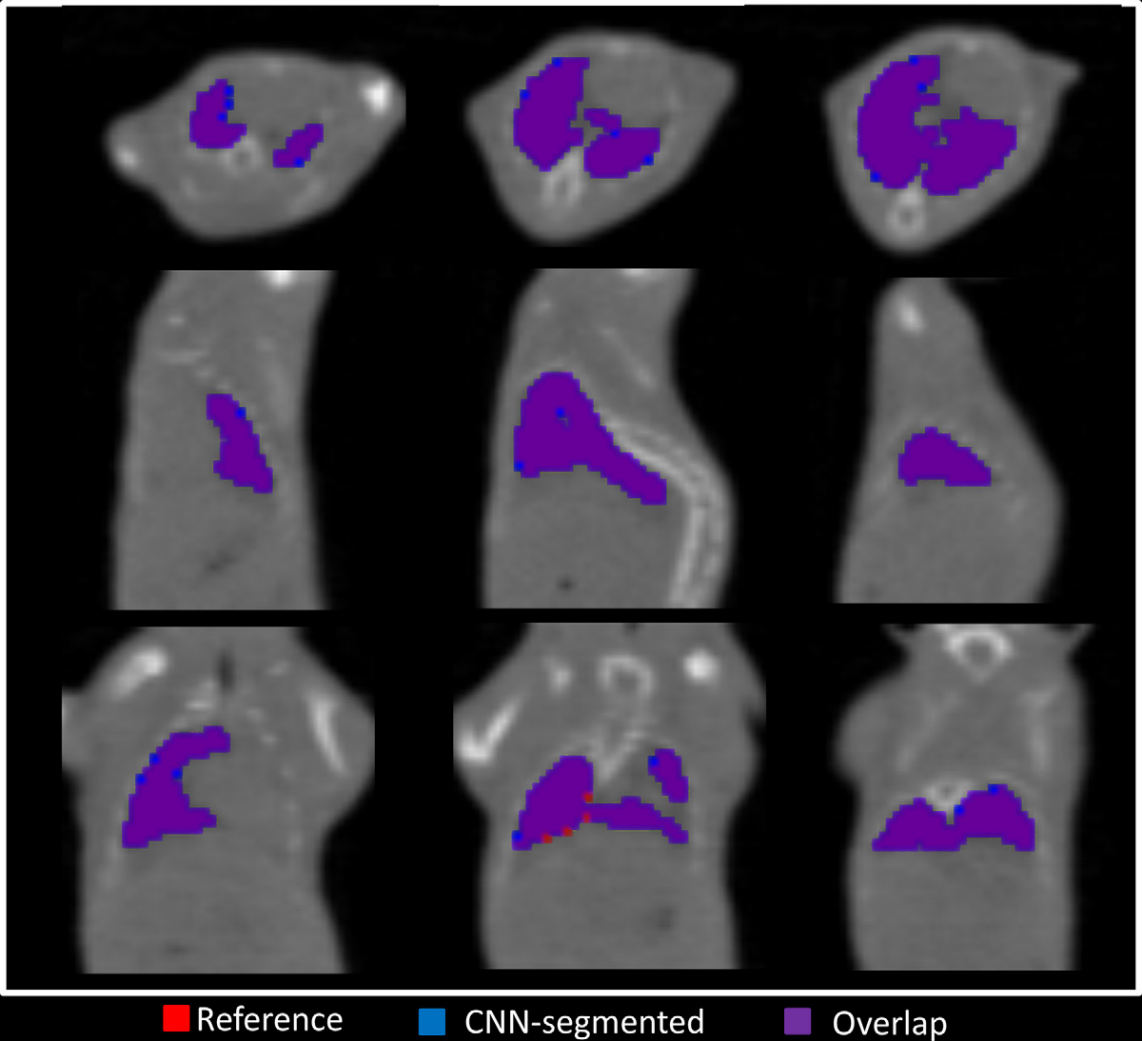
Representative segmentation results of the convolutional neural network (CNN) for a random mouse from the test set (group A). The reference lung mask (red) is overlapped with the segmented mask (blue). The intersection between the reference lung mask and segmented masks are depicted in purple.

For additional testing, the model was evaluated on an external test dataset (group B; *n* = 237 mice). Images from these mice were acquired with different resolutions as compared with those used to train the network (see Table). Observed median DSC and HD in this validation dataset were 0.966 (IQR, 0.955–0.972) and 0.31 mm (IQR, 0.30–0.32 mm), respectively (see Fig E3 [supplement]). A representative lung segmentation from the test set is shown in Figure 3; the DSC for this image was 0.981, indicating a high overlap between the reference and the CNN-segmented masks. Single discordant voxels were observed on the lung margins.

### High-Resolution Mouse Micro-CT

**Figure 4:**
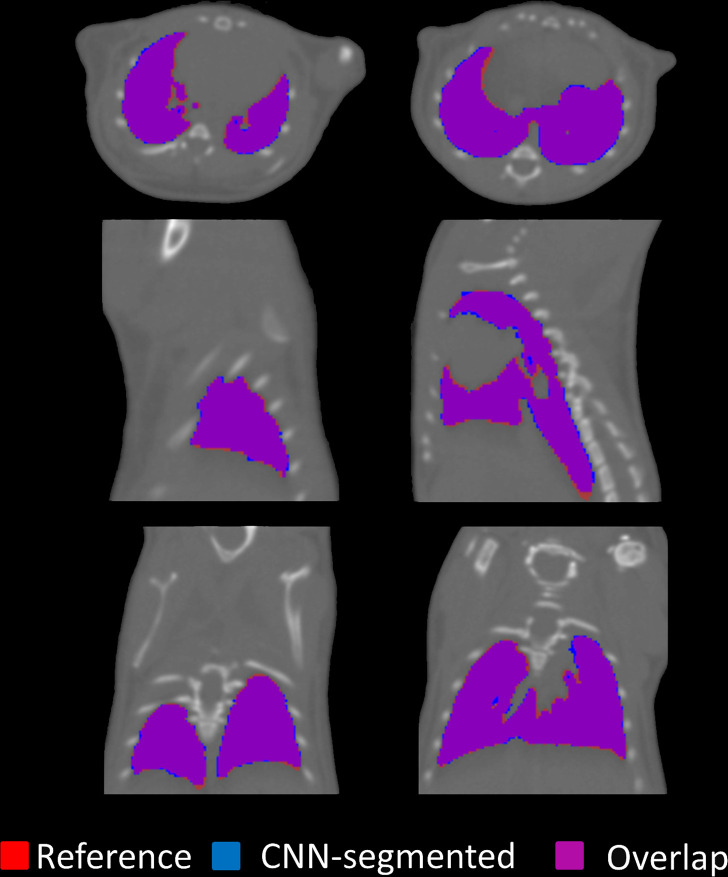
Segmentation results for a representative high-resolution mouse. The reference lung mask (red) is overlapped with the convolutional neural network (CNN)–segmented mask (blue). The intersection between the reference lung mask and segmented masks are depicted in purple.

Transfer learning findings are reported in Figure 4. The complete workflow is depicted in Figure 1 (right). Results for the high-resolution mice are shown in Figure 4, which shows a high overlap between the CNN and the semiautomatically segmented lung mask. For the four tested mice, the median DSC was 0.905 (IQR, 0.902–0.929) and the median 95th percentile of the HD was 0.33 mm (IQR, 2.61–2.78 mm).

### Spatial Mismatch Quantification

**Figure 5:**
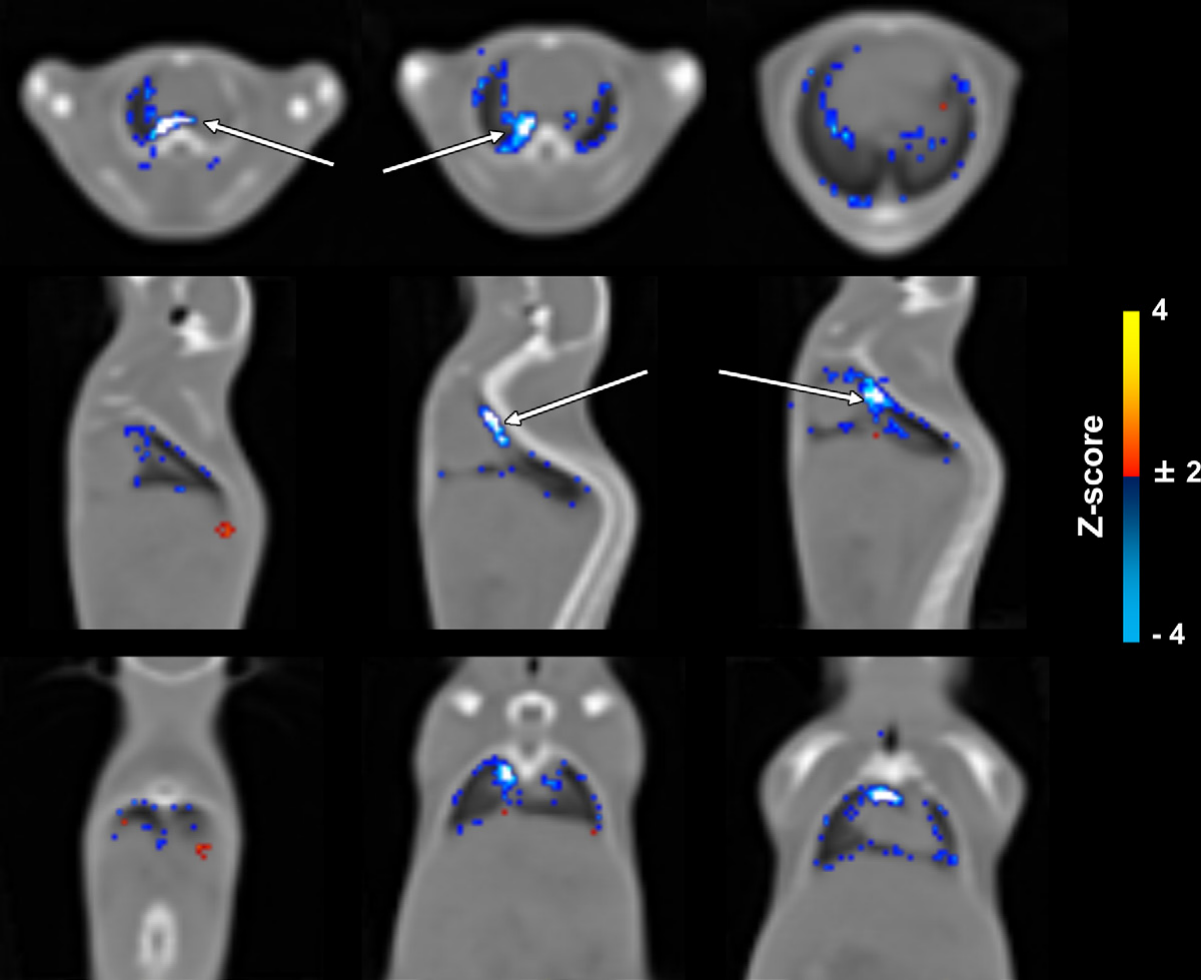
Spatial analysis of mismatches between the reference and the convolutional neural network (CNN)–segmented lung masks across the mice in the test set (group A). The voxels oversegmented by the CNN are colored blue, and the voxels undersegmented by the CNN are shown in red. Arrows = heart/lung interface, where the highest mismatch was observed.

To further evaluate the performance of the proposed approach, we aimed to highlight areas in the lungs that were more prone to diverging segmentations between CNNs and reference lung masks using data from the test set of group A (*n* = 154). Figure 5 shows three representative views of the mouse template overlays with z-scores. As can be seen, most of the voxels classified divergently were restricted to two anatomic regions (red and blue hot spots in Fig 5).

### Similarity of Approaches

**Figure 6:**
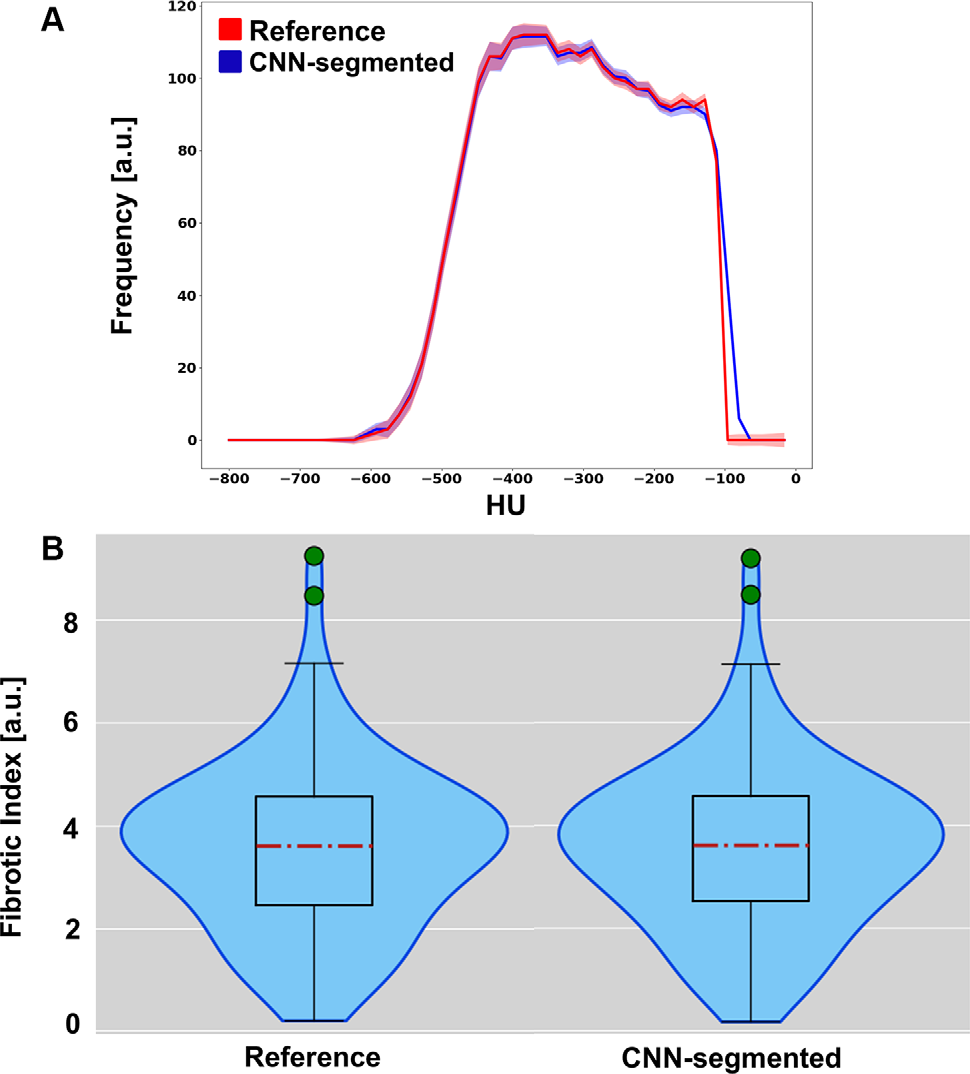
Comparison of convolutional neural network (CNN) versus reference masks using the fibrosis index and overall distributions of Hounsfield units included in masks. **(A)** Result of the histograms comparison. The histogram shows the average Hounsfield unit values across mice extracted using the semiautomatically segmented mask (reference) and is shown in red, while the histogram calculated using the CNN-segmented mask is colored blue. The shadow represents the standard error of the mean. **(B)** Violin plots show the distributions of the fibrosis indices calculated using the semiautomatically segmented lung masks (reference) and the masks segmented by the proposed method (CNN-segmented). a.u. = arbitrary unit.

Finally, data to rate similarity of semiautomatic versus CNN-based segmentation results are reported in Figure 6. Figure 6A shows the results of the gray-level histogram comparison. The two histograms showed high overlap, indicating interchangeability of the two sets of masks. Fibrosis index distributions confirmed those findings (Fig 6B).

## Discussion

In this study, we present a new method for rapid and automated lung segmentation from mouse CT scans, based on a U-Net CNN. On two test datasets, the model achieved high precision, indicated by DSCs above 0.96 and 95th percentile of the HDs below 0.5 mm. These results indicate that the CNN segmentations and reference lung masks were almost perfectly overlapping, and that there were no substantial shape mismatches.

Here, the 95th percentile of the HD was used as an evaluation metric because it is widely used in other areas of research, such as in the evaluation of brain tumor segmentation (13) and brain extraction (14). The use of this metric allowed the authors to overcome the high sensitivity of the maximum HD to outliers.

In contrast to the method developed in the study by Schoppe et al (15), in which the authors presented a deep learning–based method to segment multiple organs in healthy mice, our method can be successfully applied to segment lungs from mice with different levels of fibrosis. This method can be extremely useful in translational research, such as in the analysis of radiation-induced side effects after thoracic radiation therapy. Furthermore, it can be applied to CT images with different resolutions, and not only to high-resolution micro-CT scans. A comprehensive comparison between the proposed method and other existing approaches is presented in Table E3 (supplement).

More detailed analyses were performed to identify areas in which the CNN might yield less reliable results. Our analyses revealed that mostly voxels close to the borders of the lungs were more consistently misclassified. Of note, two hot spots of discordant segmentations were observed. In particular, the area often oversegmented by the network is close to the air-tissue interface where there is no clear separation between contiguous voxels (for example, owing to partial volume effects).

To minimize any possible registration error, a population-representing mouse template was built. This template provides a meaningful reference for the registration of mice with both fibrotic and nonfibrotic lungs. The resulting nonlinear registrations were then carefully checked, and no major failure was noticed. In contrast, selecting a random mouse from the test set as a reference yielded poor registration results, probably owing to substantial anatomic differences.

The ability of this network to segment lungs from high-resolution mouse micro-CT images was also investigated. The network was trained using a transfer learning approach on only 16 new mice. A median DSC of 0.905 was observed, which is similar to the DSC of 0.90 reported by Yan et al (5) using dynamic contrast-enhanced micro-CT images, with image resolution of 0.15 mm (isotropic), which is comparable to that of the CT images in group C (0.1 mm). The median 95th percentile HD was 0.33 mm, which is slightly higher than that for the mouse acquired with the clinical CT.

Fibrosis index and the gray-level histogram values within the lungs are often reported in studies of fibrosis development (1,2). For this reason, to justify the use of our method over semiautomatic segmentation, we extracted these two features using both masks and compared the results. Both comparisons showed high similarity, corroborating the use of this method to segment the lungs and to use the extracted mask for any further analysis.

One limitation of this study was the two-dimensional network structure used in the current approach. This architecture inherently fails to capture global context from adjacent sections, which might explain the oversegmented spots in the mismatch analysis. This issue might be at least partially solved using a three-dimensional network architecture.

Future studies will evaluate high-resolution mouse CT data, and possibly also micro-CT, in more detail, both by including additional data in the training steps as well as by evaluating lung segmentation performance in lungs with different grades of fibrosis.

In conclusion, we developed a new method for mouse lung segmentation using a CNN. This algorithm has been tested for mice with both healthy and fibrotic lungs and yielded high DSC and low HD values. The model was successfully applied to high-resolution mouse micro-CT images.
